# Supplementary material for: Risk mapping using serologic surveillance for selected One Health and transboundary diseases in Cambodian goats
Source: PLoS Negl Trop Dis. 2023 Apr 3;17(4):e0011244. doi: 10.1371/journal.pntd.0011244 (PMC10101637; doi:10.1371/journal.pntd.0011244)
Supplement: Attachment — Submitted filename: PTND-D-22-01152.docx [file pntd.0011244.s001.docx]

Thank you for giving me the opportunity to read such an interesting manuscript. I think the manuscript is well-written and overall scientifically sound, with materials and methods generally well outlined, results well described and conclusions overall well motivated. I have experience from conducting similar studies in lower-middle income countries and I am well aware of the challenges that this entails, for example in terms of study design. While random sample selection is desirable, it is often not possible in contexts such as the one where this study was conducted. I appreciate the authors’ effort to design a study with as little inherent bias as possible. My main comment on this text is however related to study design, which I think needs to be clarified somewhat. I also have some structural comments. Lastly, I think the paper would merit from more elaboration by the authors on the limitations of their study. These concerns are found among the comments below.

**Abstract**:

The abstract gives a good overview of the paper, but it is a little bit too long in my opinion and therefore, I suggest that the authors consider shortening it. Also, I am curious to know why the authors chose to emphasize the true prevalence and not the apparent prevalence, both in the abstract and results? Personally, I usually focus on apparent prevalence, and this seems to be a more common approach.

Lines 19-23: In my opinion, the introductory part of the abstract could be shortened, probably with about 70-80%.

Line 23: I believe it should be proximity, not proximately

Line 26: Was it serum samples or whole blood that you used in the analyses? Please specify

Line 28: I would rather write: “a total of 540 samples were collected from goats in six provinces and analyzed with commercially available enzyme-linked immunosorbent assays.” In my opinion, the name of the provinces is not necessary in an abstract. If the reader wants more details, he or she can read the rest of the manuscript. If you decide to keep the sentence as it is, please note that you did not perform the ELISA-kits, you performed *analyses* with the ELISA kits.

Line 29-30: Please note that you are mixing pathogens and diseases here (e.g Brucella spp. and FMD). It is better to be consistent. Please keep this in mind also in the rest of the paper.

Line 31: I suggest changing to “true seroprevalences with a 95% Confidence Interval (CI) were calculated, taking into account the usage of imperfect tests” or similar. English is not my first language but it seems like the sentence is missing something.

Line 32: I was taught that one should avoid usage of the term “risk factor”, since the variable could also be a protective factor, and because it to some readers may indicate causality (and a cross-sectional study is not enough to enable you to draw any conclusions with regards to causality). Please consider using the term “predictor variable” instead.

Line 32: I would put “calculated” instead of identified

Line 33: I assume you did multivariable analysis? If so, please add this to the sentence (or univariable logistic regression if that was the case)

Line 34: It is enough to just write “FMD” and exclude the NSP part, since you already mentioned that you have used an NSP ELISA in line 29. The assumption is that the antibody for the NSP is an antibody for FMD. Otherwise, you should also name the structure that the other ELISA’s bind to.

Lines 34-35: I would consistently use “true prevalence” instead, as frequency both can be a measure of incidence and prevalence.

Line 37: Please include OR as well for each predictor variable, and preferably also 95% confidence interval for the OR’s. Also, with such small p-values, I think it is enough to just put p<0.001.

Line 37-38: You could connect the two sentences. For example, it could be: For Q-fever, seroprevalence differed significantly between different communes (OR xx, 95% CI yy, p<0.001) and female goats were significantly more likely to be seropositive compared to male animals (OR xx, 95% CI yy, p<0.001).

Lines 38-41: Please see the above comment. Suggestion: “For FMD, seroprevalence differed significantly between communes (OR xx, 95% CI yy, p<0.001), and goats older than two years of age were significantly more likely to be seropositive compared to younger animals (OR xx, 95% CI yy, p<0.001).

Lines 41-50: Please consider to shorten this segment as well, as it is a bit long and since there is some repetition from the results above. I would suggest something similar to this “In summary, while none of the goats in the study tested positive for presence of antibodies to PPRV, the seroprevalence of FMDV was overall high, especially in older animals and in certain areas of the country. Also, while few animals were seropositive for *Brucella* spp., a higher seroprevalence of *Coxiella burnetii* was observed, especially in female animals and in some of the study areas.”

Line 50: Were the animals that you sampled all unvaccinated to all the four included diseases? If so, you could consider adding this to for example line 26 (i.e. just the word “unvaccinated”-but this is just a suggestion)

Line 50: FMD is generally considered a mild disease in small ruminants, especially in endemic areas. I am sure the virus can have a negative impact on sheep and goat production, but probably not as big as for for example cattle. However, small ruminants can act as “trojan horses” and spread the disease to more sensitive species, such as cattle, without themselves showing much clinical signs.

**Author summary**:

Line 53: Suggestion: “Smallholders often keep goats within their homesteads, which increases contact between animals and humans and thereby also the risk for zoonotic disease transmission”.

Line 54: In our study, we collected…

Line 60: I think it is enough if you either present the apparent or the true prevalence, not both. I suggest apparent prevalence.

Line 60: Why did you include n=540 here? The number of animals sampled is stated just a couple of sentences before in the text.

Line 60: “taking into account” I would say is more correct than “taken into account”

Line 65: I assume you mean that the impact of brucellosis and Q-fever on animal and human health in the areas that you have studied remain unknown, and not in general, since there is quite a lot of information on these bacteria’s impact on animals and humans. Hence, please include “in the studied areas” or similar.

Line 66-67: I feel like the last sentence is missing something. Why do you recommend research in high-risk areas? To understand why these areas are high-risk areas? Please specify.

**Introduction**

Line 69: Suggestion: “In 2018, the total number of goats in Cambodia reported by the governmental authority General Directorate of Animal Health and Production (GDAHP), was…”

Line 72: …reported that in 2019**,** Cambodia … (please include a comma)

Line 73: Is there any informal trade with neighboring countries that would not appear in these numbers?

Line 74: “given goats to farmers” is probably more accurate

Line 75: Just out of curiosity: why only a secondary source of income? Could it not also be a household’s primary source of income? Do they always have other primary sources of livelihood?

Line 75: “…for **the** farmers who raise them…

Lines 78-80: Cambodia is repeated quite a few times, maybe replace at least one of them with “the country” or similar?

Line 85: Small animals to many westerners means dogs and cats. I would therefore instead write “smallholder livestock farmers” or similar.

Line 86: Consider exchanging household to homestead

Line 86: I am not familiar with the term “One Health pathogen”. Therefore, I would probably instead write “zoonotic pathogens”.

Line 86-94: This text segment is a bit confusing to me and could be clarified somewhat. I suggest something like this:

There is hence a considerable risk for spread of transboundary diseases, both between animals as well as between animals and humans. However, available information on goat health and production in Cambodia is limited [16]. Previous studies of Cambodian goats mostly focused on forages, nutrition, and parasite burdens and control [5, 17-19]. In neighboring countries, pathogens such as Coxiella burnetii (causative agent of Q-fever) (reference), Brucella spp (causative agent of brucellosis) (ref) and FMD are present., and introduction of these pathogens into the Cambodian goat population could have adverse impacts on small ruminant production in the country. There was no publication on disease surveillance programs in Cambodian goats at the time of our study (ref). Thus, an active…

Line 96: I would write NAHPRI **and** GDP, in collaboration…

Line 99: Since your study does not intend to meet all the objectives of the program, I would include something like this: “This study aims to contribute with information on the seroprevalence of FMDV, PPRV, *Brucella* spp., and *Coxiella burnetii* to the larger program. Furthermore, this study aims to investigate associations between selected predictor variables and seroprevalence”… or similar.

**Materials and methods**

Line 110: Do you know why the provinces no longer raised goats? Did the farmers choose to stop for some reason? If yes, it might be interesting to know the reason why, as it potentially could be due to high prevalence of disease or similar

Line 115: Again, do you know why the number had decreased so drastically?

Lines 116-118: I am not an expert so please correct me if I am wrong, but since you also chose animals within herds (and not just communes and herds), isn’t it more correct to say that you did a multi-stage survey?

Lines 116-118: I suggest that the authors include a sentence that highlights the fact that the selection of provinces, communes and villages were non-random. This can be deduced from the rest of the text but I do feel that this should be clearly stated for the reader.

Lines 116-118: How did you choose the number of communes, villages and herds? How many herds per villages and animals per herd were sampled? Was the selection of animals and herds random?

Line 144: Which Brucella subspecies does your ELISA detect antibodies to? Melitensis? Abortus? Suis? Ovis? Please include this information in the text

Lines 118-123: Can you please motivate the choice of these values, at least for some of the parameters (for example design prevalence and cluster size).

Did you make a directed acyclic graph (DAG) prior to analysis, to for example identify confounders?

**Results**

Overall, I suggest that the authors consider to rewrite the results in subsections, for example descriptive statistics in one subsection, seroprevalence result in one subsection and the predictor variable analysis in one subsection. Furthermore, I cannot find tables where the predictor variables are presented? Please present this information also in table form, not just in the text.

Line 171: I think it should be figure 3.

Line 174: You should include the fact that you did body condition scoring in the materials and methods section as well, along with a description of how or at least a reference to a BCS scoring manual. Especially since BCS in goats generally is considered more tricky compared to in for example sheep and cattle.

Line 176: When were the sheep and goats last vaccinated for FMD?

Lines 178-181: Please state apparent prevalence as well, and include confidence interval both for the apparent and true prevalence. Also, similar to above, I am curious to know why the authors chose to emphasize the true rather than apparent prevalence.

Lines 178-187: It might be confusing for the reader when you jump between pathogens in your description of the seroprevalence results. Please consider separating them so that you first describe one pathogen, then another etc.

Lines 188-198: For really low p-values, please write instead p<0.001 or similar. Furthermore, some of the results are repeated, only with a little bit more information the second time, for example predictor variables associated with FMD seroprevalence. You can include all the information once, to avoid repetition. I gave suggestions for how to do this in my comments on the abstract.

Table 2: Please include apparent seroprevalence in the table as well

**Discussion**

The discussion is in my opinion a bit difficult to follow and its missing a guiding red thread. It contains a lot of text that would be better placed in the introduction than the discussion, especially in the first text segment (lines 207-226). Some of the information might not need to be included at all. Please keep in mind that the discussion is meant to summarize your findings, interpret them and discuss them within the context of previous studies, as well as to discuss limitations of your study and give recommendations for future. Similarly to in the results, it would be easier for the reader if you present the results separately for each pathogen, so that you do not jump back and forth so much. Lastly, and perhaps most importantly, what are the study limitations? For example limitations with regards to your choice of study design, etc?

Lines 227: I suggest: “Our data revealed that almost half of the **sampled** goats…”

Line 239: Please discuss the fact that some of the sampled goats were vaccinated for FMD and how you suspect this might have affected the results. The ID-Vet FMD NSP ELISA is a DIVA test (when purified vaccines are used) according to the manufacturer.

Line 247: Can you really make this statement with absolute certainty? Five animals did after all test positive, and I do not think this result can be completely discarded just because the test you used is not perfect. I would rather discuss this finding and say that the fact that only five individuals tested positive and that the true prevalence is zero indicates that PPRV is not circulating in the goat population, but more research is needed on the subject.

Lines 252-255: While I see your point with regards to maternal antibodies, I do not completely understand the conclusion related to number of previous vaccinations. The ELISA that you have used is described as a DIVA test by the manufacturer, and should only detect antibodies deriving from a natural infection.

Lines 266-267: More correct would probably be: “This may be caused by the imbalanced sex proportion…”

Line 268: “…goat production appears to **have** potential”, or similar.

Line 270: smallholder farmers, not small farmers

Line 271: I would leave nutrition out since you have not looked at that

Lines 272-273: Some of the pathogens appear to be circulating in the population based on the seroprevalence results, but not all (PPR for example). Please rephrase.
