# Supplementary material for: Risk mapping using serologic surveillance for selected One Health and transboundary diseases in Cambodian goats
Source: PLoS Negl Trop Dis. 2023 Apr 3;17(4):e0011244. doi: 10.1371/journal.pntd.0011244 (PMC10101637; doi:10.1371/journal.pntd.0011244)
Supplement: Attachment — Submitted filename: PTND-D-22-01152_rebuttal_SDB.docx [file pntd.0011244.s002.docx]

# In this manuscript, Siengsanan-Lamont and co-authors describe the seroprevalence of antibodies against a variety of pathogens in goats in Cambodia. The reviewers felt that the study was largely well conducted and solid scientifically. There were a number of points they raised that need to be addressed prior to publication: - There is not sufficient description of study limitation in the Discussion section.

# ***Response:*** *more detail were added to the discussion section.*

# - The Methods are lacking some key details about the ELISAs used in the study, such as the sensitivity and specificity and the specific antigenic targets.

# ***Response:*** *the citations of the test sensitivity and specificity are provided as they were previously published.*

# - The authors should describe seroprevalence in terms of antibodies against the pathogens, not in terms of disease (e.g. PPRV not PPR; C. burnetii not Q fever; etc).

***Response:*** *amended as suggested*

- The authors should address any impact of FMDV vaccination on the results.
***Response:*** *amended as suggested*

# - The Abstract is much longer than it needs to be. I would put this one as optional, but I think it would be a stronger publication if the Abstract were more concise and clearer. I would say the same about the Author Summary.

***Response:*** *amended as suggested*

# - The Methods do not describe how the specific goat herds were selected, and I don't believe there was any statistical adjustment for clustering.

***Response:*** *amended as suggested; more detail were added.*

# - Data availability, per PLOS policy, is not sufficiently addressed. There is no compelling statement on why these data can't be shared, and just mentioning to ask a government agency without even providing contact information does not address the requirement.

***Response:*** *amended as suggested; contact detail was added.*

# Reviewer 1

Thank you for giving me the opportunity to read such an interesting manuscript. I think the manuscript is well-written and overall scientifically sound, with materials and methods generally well outlined, results well described and conclusions overall well motivated. I have experience from conducting similar studies in lower-middle income countries and I am well aware of the challenges that this entails, for example in terms of study design. While random sample selection is desirable, it is often not possible in contexts such as the one where this study was conducted. I appreciate the authors' effort to design a study with as little inherent bias as possible. My main comment on this text is however related to study design, which I think needs to be clarified somewhat. I also have some structural comments. Lastly, I think the paper would merit from more elaboration by the authors on the limitations of their study. These concerns are found among the comments below.

**Abstract**:

The abstract gives a good overview of the paper, but it is a little bit too long in my opinion and therefore, I suggest that the authors consider shortening it. Also, I am curious to know why the authors chose to emphasise the true prevalence and not the apparent prevalence, both in the abstract and results? Personally, I usually focus on apparent prevalence, and this seems to be a more common approach.

**Response:** *thank you very much for your kind words and understanding of the limitations facing the study. One of our government partner's objectives was to prove freedom from PPR, which was the main reason we chose to present the true prevalence over the apparent prevalence. Regarding the abstract, the authors deleted a background sentence to shorten the section as suggested.*

Lines 19-23: In my opinion, the introductory part of the abstract could be shortened, probably with about 70-80%.

***Response:*** *amended as suggested*

Line 23: I believe it should be proximity, not proximately

***Response:*** *amended as suggested*

Line 26: Was it serum samples or whole blood that you used in the analyses? Please specify

***Response:*** *amended as suggested*

Line 28: I would rather write: "a total of 540 samples were collected from goats in six provinces and analysed with commercially available enzyme-linked immunosorbent assays." In my opinion, the name of the provinces is not necessary in an abstract. If the reader wants more details, he or she can read the rest of the manuscript. If you decide to keep the sentence as it is, please note that you did not perform the ELISA-kits, you performed *analyses* with the ELISA kits.

***Response:*** *amended as suggested*

Line 29-30: Please note that you are mixing pathogens and diseases here (e.g Brucella spp. and FMD). It is better to be consistent. Please keep this in mind also in the rest of the paper.

***Response:*** *amended as suggested*

Line 31: I suggest changing to "true seroprevalences with a 95% Confidence Interval (CI) were calculated, taking into account the usage of imperfect tests" or similar. English is not my first language but it seems like the sentence is missing something.

***Response****: The sentence was amended to "True seroprevalences with a 95% Confidence Interval (CI) taking into account imperfect tests, Risk factors and Odds ratios (ORs) were calculated to better understand the disease distribution and epidemiology".*

Line 32: I was taught that one should avoid usage of the term "risk factor", since the variable could also be a protective factor, and because it to some readers may indicate causality (and a cross-sectional study is not enough to enable you to draw any conclusions with regards to causality). Please consider using the term "predictor variable" instead.

***Response****: As the significant ORs are greater than 1, indicating the positive correlation, these are risk factors, not protective factors.*

Line 32: I would put "calculated" instead of identified

***Response:*** *amended as suggested*

Line 33: I assume you did multivariable analysis? If so, please add this to the sentence (or univariable logistic regression if that was the case)

***Response:*** *Only the FMD and Q fever datasets were fitted with multivariable logistic regression models. The Brucellosis and PPR were not as none of the univariate models were significant.*

Line 34: It is enough to just write "FMD" and exclude the NSP part, since you already mentioned that you have used an NSP ELISA in line 29. The assumption is that the antibody for the NSP is an antibody for FMD. Otherwise, you should also name the structure that the other ELISA's bind to.

***Response:*** *amended as suggested*

Lines 34-35: I would consistently use "true prevalence" instead, as frequency both can be a measure of incidence and prevalence.

***Response:*** *amended as suggested*

Line 37: Please include OR as well for each predictor variable, and preferably also 95% confidence interval for the OR's. Also, with such small p-values, I think it is enough to just put p<0.001.

***Response:*** *Only significant ORs are presented to avoid misinterpretation.*

*For those variables that were tested significate in univariate analysis but OR was not significant, the interpretation is that there are some significant differences between groups within the variable, but none of these groups has a significant OR.*

Line 37-38: You could connect the two sentences. For example, it could be: For Q-fever, seroprevalence differed significantly between different communes (OR xx, 95% CI yy, p<0.001) and female goats were significantly more likely to be seropositive compared to male animals (OR xx, 95% CI yy, p<0.001).

***Response:*** *As described in the above response, only significant ORs are presented in the paper. To avoid confusion, the sentences are separated.*

Lines 38-41: Please see the above comment. Suggestion: "For FMD, seroprevalence differed significantly between communes (OR xx, 95% CI yy, p<0.001), and goats older than two years of age were significantly more likely to be seropositive compared to younger animals (OR xx, 95% CI yy, p<0.001).

***Response:*** *As described in the above response, only significant ORs are presented in the paper. To avoid confusion, the sentences are separated.*

Lines 41-50: Please consider to shorten this segment as well, as it is a bit long and since there is some repetition from the results above. I would suggest something similar to this "In summary, while none of the goats in the study tested positive for presence of antibodies to PPRV, the seroprevalence of FMDV was overall high, especially in older animals and in certain areas of the country. Also, while few animals were seropositive for *Brucella* spp., a higher seroprevalence of *Coxiella burnetii* was observed, especially in female animals and in some of the study areas."

***Response:*** *The sentences were amended .*

Line 50: Were the animals that you sampled all unvaccinated to all the four included diseases? If so, you could consider adding this to for example line 26 (i.e. just the word "unvaccinated"-but this is just a suggestion)

***Response:*** *Some of the animals were vaccinated against FMD and HS (as shown in Table 2).*

Line 50: FMD is generally considered a mild disease in small ruminants, especially in endemic areas. I am sure the virus can have a negative impact on sheep and goat production, but probably not as big as for for example cattle. However, small ruminants can act as "trojan horses" and spread the disease to more sensitive species, such as cattle, without themselves showing much clinical signs.

***Response:*** *Agreed.*

**Author summary**:

Line 53: Suggestion: "Smallholders often keep goats within their homesteads, which increases contact between animals and humans and thereby also the risk for zoonotic disease transmission".

***Response:*** *Amended as suggested*

Line 54: In our study, we collected…

***Response:*** *Amended to "this study collected…"*

Line 60: I think it is enough if you either present the apparent or the true prevalence, not both. I suggest apparent prevalence.

***Response:*** *Amended as suggested*

Line 60: Why did you include n=540 here? The number of animals sampled is stated just a couple of sentences before in the text.

***Response:*** *Amended as suggested (the N was deleted).*

Line 60: "taking into account" I would say is more correct than "taken into account"

***Response:*** *Amended as suggested*

Line 65: I assume you mean that the impact of brucellosis and Q-fever on animal and human health in the areas that you have studied remain unknown, and not in general, since there is quite a lot of information on these bacteria's impact on animals and humans. Hence, please include "in the studied areas" or similar.

***Response:*** *Amended as suggested*

Line 66-67: I feel like the last sentence is missing something. Why do you recommend research in high-risk areas? To understand why these areas are high-risk areas? Please specify.

***Response:*** *Amended to "Thus, future investigations targeting high-risk areas would be recommended to better understand the disease epidemiology and their impacts on human and animal health.".*

**Introduction**

Line 69: Suggestion: "In 2018, the total number of goats in Cambodia reported by the governmental authority General Directorate of Animal Health and Production (GDAHP), was…"

***Response:*** *Amended to "In 2018, the total number of goats in Cambodia reported by the General Directorate of Animal Health and Production (GDAHP) was".*

Line 72: …reported that in 2019**,** Cambodia … (please include a comma)

***Response:*** *Amended as suggested*

Line 73: Is there any informal trade with neighboring countries that would not appear in these numbers?

***Response:*** *there was no report on informal trade with neighbouring countries.*

Line 74: "given goats to farmers" is probably more accurate

***Response:*** *Amended as suggested*

Line 75: Just out of curiosity: why only a secondary source of income? Could it not also be a household's primary source of income? Do they always have other primary sources of livelihood?

***Response:*** *Amended to "While goats provide meat for family consumption, they are also considered an additional source of income for the family.".*

Line 75: "…for **the** farmers who raise them…

***Response:*** *Amended to "While goats provide meat for family consumption, they are also considered an additional source of income for the family.".*

Lines 78-80: Cambodia is repeated quite a few times, maybe replace at least one of them with "the country" or similar?

***Response:*** *Amended as suggested*

Line 85: Small animals to many westerners means dogs and cats. I would therefore instead write "smallholder livestock farmers" or similar.

***Response:*** *Amended as suggested*

Line 86: Consider exchanging household to homestead

***Response:*** *the "homestead" may not be suitable in this context as it gives a sense of a bigger land.*

Line 86: I am not familiar with the term "One Health pathogen". Therefore, I would probably instead write "zoonotic pathogens".

***Response:*** *The authors would like to keep the "One Health pathogen" as it is the same word used for the topic of the article.*

Line 86-94: This text segment is a bit confusing to me and could be clarified somewhat. I suggest something like this:

There is hence a considerable risk for spread of transboundary diseases, both between animals as well as between animals and humans. However, available information on goat health and production in Cambodia is limited [16]. Previous studies of Cambodian goats mostly focused on forages, nutrition, and parasite burdens and control [5, 17-19]. In neighboring countries, pathogens such as Coxiella burnetii (causative agent of Q-fever) (reference), Brucella spp (causative agent of brucellosis) (ref) and FMD are present., and introduction of these pathogens into the Cambodian goat population could have adverse impacts on small ruminant production in the country. There was no publication on disease surveillance programs in Cambodian goats at the time of our study (ref). Thus, an active…

***Response:*** *Some of these pathogens are considered endemic in the country despite no information on the disease in Cambodian goats. The authors suspected that the goat populations had already been exposed to the diseases prior to our study. The information about the neighbouring countries was to state that Cambodia could be in a similar situation (which was our hypothesis). The authors did not suggest that the diseases would be introduced via transboundary routes.*

Line 96: I would write NAHPRI **and** GDP, in collaboration…

***Response:*** *NAHPRI is one of the departments within GDAHP. The program collaboration detail was omitted (only included in the acknowledgement section) to shorten the introduction section as previously suggested.*

Line 99: Since your study does not intend to meet all the objectives of the program, I would include something like this: "This study aims to contribute with information on the seroprevalence of FMDV, PPRV, *Brucella* spp., and *Coxiella burnetii* to the larger program. Furthermore, this study aims to investigate associations between selected predictor variables and seroprevalence"… or similar.

***Response:*** *the sentences were amended to" Thus, this study's main objectives were to enhance the animal disease diagnostic capacity through practising, estimate the prevalence of priority diseases in Cambodian goats (including FMD, brucellosis and Q fever), and provide scientific evidence of Peste des Petits Ruminants (PPR) freedom.".*

**Materials and methods**

Line 110: Do you know why the provinces no longer raised goats? Did the farmers choose to stop for some reason? If yes, it might be interesting to know the reason why, as it potentially could be due to high prevalence of disease or similar

***Response:*** *the* *provincial staff told us that the farmers sold off their goats but for no specific reason.*

Line 115: Again, do you know why the number had decreased so drastically?

***Response:*** *authors did not have the information.*

Lines 116-118: I am not an expert so please correct me if I am wrong, but since you also chose animals within herds (and not just communes and herds), isn't it more correct to say that you did a multi-stage survey?

***Response:*** *correct, it is a multi-stage survey (same as a 2-stage survey, however it is the name the Epitools website used).*

Lines 116-118: I suggest that the authors include a sentence that highlights the fact that the selection of provinces, communes and villages were non-random. This can be deduced from the rest of the text but I do feel that this should be clearly stated for the reader.

**Response*:*** *the sentences were amended to Line:102-107 and 118-121 "The selection criteria of the targeted provinces were based on 1) high numbers of goats and 2) high activity of goat meat consumption, while for communes and villages were randomly selected.". However, during the sample collection, some randomly selected villages had no or low numbers of goats. Thus, the communes and villages included in this study were based on the available goat population and convenient access.*

Lines 116-118: How did you choose the number of communes, villages and herds? How many herds per villages and animals per herd were sampled? Was the selection of animals and herds random?

***Response:*** *The village comprises a small number of households which was considered as a herd (as animals often share the grazing patches).*

Line 144: Which Brucella subspecies does your ELISA detect antibodies to? Melitensis? Abortus? Suis? Ovis? Please include this information in the text

***Response:*** *amended as suggested (Line 143)*

Lines 118-123: Can you please motivate the choice of these values, at least for some of the parameters (for example design prevalence and cluster size).

Did you make a directed acyclic graph (DAG) prior to analysis, to for example identify confounders?

***Response:*** *amended as suggested, more detail added Line111-116*

**Results**

Overall, I suggest that the authors consider to rewrite the results in subsections, for example descriptive statistics in one subsection, seroprevalence result in one subsection and the predictor variable analysis in one subsection. Furthermore, I cannot find tables where the predictor variables are presented? Please present this information also in table form, not just in the text.

Line 171: I think it should be figure 3.

***Response:*** *table is added as suggested.*

Line 174: You should include the fact that you did body condition scoring in the materials and methods section as well, along with a description of how or at least a reference to a BCS scoring manual. Especially since BCS in goats generally is considered more tricky compared to in for example sheep and cattle.

***Response:*** *The following sentences were added to Line 128-130" Animal information, including age, sex, vaccination history and body condition score (BCS) was collected using a sample collection form accompanying animal specimens. The BCS used for our study was score 1 (lean) to 5 (fat).". The staff who collected the samples and data were well-trained central lab staff.*

Line 176: When were the sheep and goats last vaccinated for FMD?

***Response:*** *authors did not have the information.*

Lines 178-181: Please state apparent prevalence as well, and include confidence interval both for the apparent and true prevalence. Also, similar to above, I am curious to know why the authors chose to emphasise the true rather than apparent prevalence.

***Response:*** *the values are provided in Table 2. One of the main objectives was to prove PPR disease freedom which is why the authors chose the true prevalence. Apparent prevalence can be easily calculated using the figures provided in Table 2.*

Lines 178-187: It might be confusing for the reader when you jump between pathogens in your description of the seroprevalence results. Please consider separating them so that you first describe one pathogen, then another etc.

***Response:*** *Same figures are shown in Table 2.*

Lines 188-198: For really low p-values, please write instead p<0.001 or similar. Furthermore, some of the results are repeated, only with a little bit more information the second time, for example predictor variables associated with FMD seroprevalence. You can include all the information once, to avoid repetition. I gave suggestions for how to do this in my comments on the abstract.

Table 2: Please include apparent seroprevalence in the table as well

***Response:*** *P-values were amended as suggested. As described above, some sentences can not be combined together as they are outcomes from a different analysis. The apparent prevalences are presented in table 2.*

**Discussion**

The discussion is in my opinion a bit difficult to follow and its missing a guiding red thread. It contains a lot of text that would be better placed in the introduction than the discussion, especially in the first text segment (lines 207-226). Some of the information might not need to be included at all. Please keep in mind that the discussion is meant to summarise your findings, interpret them and discuss them within the context of previous studies, as well as to discuss limitations of your study and give recommendations for future. Similarly to in the results, it would be easier for the reader if you present the results separately for each pathogen, so that you do not jump back and forth so much. Lastly, and perhaps most importantly, what are the study limitations? For example limitations with regards to your choice of study design, etc?

***Response:*** *As suggested****,*** *the 1st paragraph of the discussion section was reorganised to address the limitation of the estimated goat population, site selection and study design. Less relevant sentences were excluded. The rest of the paragraphs compared study results with other previous studies.*

Lines 227: I suggest: "Our data revealed that almost half of the **sampled** goats…"

***Response:*** *amended as suggested*

Line 239: Please discuss the fact that some of the sampled goats were vaccinated for FMD and how you suspect this might have affected the results. The ID-Vet FMD NSP ELISA is a DIVA test (when purified vaccines are used) according to the manufacturer.

***Response:*** *amended as suggested (Lines:268-270)*

Line 247: Can you really make this statement with absolute certainty? Five animals did after all test positive, and I do not think this result can be completely discarded just because the test you used is not perfect. I would rather discuss this finding and say that the fact that only five individuals tested positive and that the true prevalence is zero indicates that PPRV is not circulating in the goat population, but more research is needed on the subject.

***Response:*** *amended to" As the PPRV true seroprevalence and 95% CIs were zero or near zero, there was no evidence of PPR in this study."*

Lines 252-255: While I see your point with regards to maternal antibodies, I do not completely understand the conclusion related to number of previous vaccinations. The ELISA that you have used is described as a DIVA test by the manufacturer, and should only detect antibodies deriving from a natural infection.

***Response:*** *amended to" The older the animals are, the more likely the animals are to be vaccinated multiple times (increasing chances of exposure to an unpurified vaccine) or exposed to FMD viruses,..."*

Lines 266-267: More correct would probably be: "This may be caused by the imbalanced sex proportion…"

***Response:*** *amended as suggested*

Line 268: "…goat production appears to **have** potential", or similar.

***Response:*** *amended as suggested*

Line 270: smallholder farmers, not small farmers

***Response:*** *amended as suggested*

Line 271: I would leave nutrition out since you have not looked at that

***Response:*** *amended as suggested*

Lines 272-273: Some of the pathogens appear to be circulating in the population based on the seroprevalence results, but not all (PPR for example). Please rephrase.

***Response:*** *amended as suggested*

Some other minor comments:

- Abstract, line 32: "odds" in "odds ratios" does not need to be capitalised, and probably best described as "calculated" or "estimated" not "identified"

***Response:*** *amended as suggested*

- Abstract, line 35: Looks like there is a typo in the confidence interval for FMD NSP.

***Response:*** *amended as suggested*

- Abstract: I don't understand this statement "Q fever seroprevalence of female goats was significantly higher than males; however, it may be due to an imbalance between male and female populations." What "imbalance"? Would this have been addressed by statistical adjustments?

***Response:*** *amended to"* to imbalanced male and female populations."

- Abstract: Why is FMD identified by the NSP when the other pathogens the antigen is not mentioned?

***Response:*** *as discussed in the manuscript, antibodies against NSP are used to differentiate natural infection-induced antibodies.*

- Author Summary, lines 59-60: The framing of "true seroprevalence" is confusing.

***Response:*** *amended as suggested*

- Similarly in the Results, line 180: How can a true prevalence be "less than zero"? I think that's part of the problem I have with this interpretation. So, the assumption is that the few positives are false-positives, which may very well be true, it's just hard to accept outright that the 95% CI is bounded by 0.0 when there were samples that tested positive.

***Response:*** *amended to"...was zero".*

- Discussion, line 277: "Our study provided scientific evidence to support PPR freedom." Is this meant to convey that they study supports the idea that these regions are free of PPRV? I understand that the tool you used is not sufficiently specific, but then those 5 positives need to be better characterised if you are trying to demonstrate absence. I think it's fair say that PPRV is low to non-existent but more specific assays are needed, or something less concrete that is consistent with the data.

**Response:** *PPR is a severe disease, and the fact that there was no previous report of clinical disease in the country. The calculation suggested that the 5 positives would be fault positives due to the imperfect test. The following sentences were added to Line 280- 285 "Our study revealed that PPR true seroprevalence and 95% CIs were zero or near zero. PPR is a severe viral disease with morbidity and mortality rates of up to 90% and 40%, respectively [44]. As there was no previous report of PPR in the country prior to the time of this study, our study provided scientific evidence to support PPR freedom. However, Thailand reported the first detection of PPR in goats imported from West Africa on February 2021 [45]. More studies should be conducted to monitor the extent of the PPR introduction to the Southeast Asia region ".*

- Discussion, lines 268-271: The statement about the future of goat production is unrelated to the current study. I recommend you keep your final conclusions tied to the current study.

***Response:*** *amended as suggested*

# Reviewer 2

This is a well-conducted study with clear results which are well reported. There are no obvious scientific flaws in the work.
Line 136-7. Centrifugal force should always be expressed as "x g", not "revolutions per minute", as the force will vary according to the diameter of the centrifuge rotor as well as the rotational speed.

***Response:*** *amended as suggested*

Yes, with the exception of Table 1 which I found unclear as to which "Districts/Municipalities" belonged to which "Province". Maybe the demarcations should be clearer with the Province" name at the very top of the column.

***Response:*** *amended as suggested*

Line 180. The true prevalence cannot be "less than zero" ! It is zero.

***Response:*** *amended as suggested*

I wonder if Figure 4 (%Brucellosis seroprevalence) has any validity with only 2 positive samples in the whole study ! I would leave it out of the paper.

***Response:*** *amended as suggested*

# Reviewer 3

Abstract
L23: …..‘close proximity’;

***Response:*** *amended as suggested*

L28: ….were used to detect……;

***Response:*** *amended as suggested by another reviewer*

L31/32: Risk factors were identified using odds ratios;

***Response:*** *amended as suggested by another reviewer*

L31-33: categorise the variables into dependent and independent variables;

***Response:*** *amended as suggested*

L34: Say Brucella species rather than Brucella spp.;

***Response:*** *amended as suggested*

L35: There is possibly an error in the CI for 7.2%;

***Response:*** *amended as suggested*

L37 and throughout the manuscript: when the p-value is very small, it is expressed as
p<0.001 or p<0.0001 depending on the significance levels desired;

***Response:*** *amended as suggested*

L39: Only age, more than…. years was a significant risk factor……;

***Response:*** *amended as suggested*

L39-41: For FMD NSP, why isn't commune a significant risk factor when p=2.2x10-16 or rephrase what you have said to make it clear;

***Response:*** *Both (age and commune) are significant risk factors (significant p-value). However, only one age group has a significate OR against the reference group.*

L42/43: Correct to: Q fever seroprevalence for female goats was significantly higher than for males. Why would an imbalance between male and female populations affect the sero-prevalence?-see also L266-267;

***Response:*** *thank you very much for the comment.* *The following sentences were added to Line 259" Seroprevalence of Q fever in female goats were significantly higher than in male. C. Burnetii causes reproductive losses (abortion and stillborn) in female goats where the bacteria are highly concentrated in the body and amniotic fluid of aborting animals. Goats sold for meat were likely to be young males (except those kept for breeding) which were less likely to be exposed to the bacteria during their lifetime compared to female goats.*

L49: It shouldn't be that animals should be vaccinated on evidence from seroprevalence. Evidence of actual disease signs or virus detection is better proof of disease presence;

***Response:*** *Agree, However, there were no other goat disease studies in Cambodia. As seroprevalence is high, vaccination is a recommended control measure in endemic countries by WOAH.*

L…- L… isn't the author summary the same as the abstract?;

***Response:*** *The section was required by the journal.*

Introduction
L78: Replace 'nowadays' with 'currently';

***Response:*** *amended as suggested*

L80: …as local production is still low (remove still in short supply);

***Response:*** *amended as suggested*

L86: Moreover, some health pathogens reported……..;

***Response:*** *amended as suggested*

L99-100: Include PPR since you investigated it as well;

***Response:*** *PPR has not been reported in the country. PPR was recently reported for the first time in Thailand (imported animals) in 2021 which was after our study.*

L103: Put a full stop at the end of the sentence;

***Response:*** *amended as suggested*

The objective of the study is clearly articulated. The study design is appropriate to address the stated objectives. The study population is clearly described and appropriate for the hypothesis being tested. The sample size is sufficient to ensure adequate power to address the hypothesis being tested. The statistical analyses used are inadequate to support conclusions. Ethical and regulatory requirements have been met.

L116-117: Are you demonstrating sero-prevalence of the diseases or freedom from the diseases/ the two objectives result in very different sample sizes. Also, you were investigating four diseases. The limits of the parameters used for sample size calculation were for which lead disease and tests? And why?

***Response:*** *Authors designed the study with the objective of proving PPR freedom. However, we also tested for other diseases too. Limitations of the design are discussed in the discussion (more detail added).*

It is better if the figures and the table are separated by the sections of text on pg. 3 as appropriate. For instance, have Figure 1 immediately after explaining what it is about, then continue with text and place the table and Figure 2 after the second bit of text.

***Response:*** *amended as suggested*

L117-118: It is not clear what this sentence means. Also state clearly what your sampling method was;

**Response:** the "2-Stage surveys for demonstration of freedom" is the name of the technique used for calculation, see https://epitools.ausvet.com.au/twostagefreedomss

L116-122: Give the formula that Epitools utilised and explain the parameters in the formula followed by the limits you used (which you have described) and why. Give citations/references as well;

***Response:*** *the formula is described in the reference provided [20].*

L121-122….sample size of 30 goats per village was calculated….But then according to Table 1, some villages had sample sizes <30;

***Response****: authors* *restructured the sample size calculation section and added more detail line 101-120.*

L122-123: place this sentence in the results section;

***Response:*** *amended as suggested*

L135: But direct contact with icepacks could cause haemolysis of whole blood. How did you prevent this?;

***Response****: the tubes usually sit upright in a rack inside a ziplock bag. Detail is added to line 131.*

L154: These cutoffs have some overlap;

***Response:*** *amended as suggested*

L155: These parameters/limits are more important in the section where you calculate the sample size;

***Response:*** *amended as suggested more detail added*

L157/58: Give the formula used, the parameters used and their limits, why and the appropriate references. As in 116-122, even if a tool is used, there is an underlying formula;

***Response****: Thank you for this comment.*

L163: Given that you have more or less used cluster sampling technique, A Generalised Linear Mixed regression Model (GLMM) would have been more appropriate for your analysis so as to include random and fixed effects;

***Response****: authors used GLM with all fixed effects.*

In Figure 2, show where Cambodia is located globally then zoom out the study areas, making clear the link between the names in the legend and those in the map and text;

***Response:*** *thank you for the suggestion. We are waiting for the revision from the graphic designer, and these will be included in the final version of the manuscript.*

You could present results on herd structure (age, sex) and husbandry characteristics (vaccination, grazing) of the study animals before presenting the test results) to justify some of the discussions in the discussion section;
Instead of n=540; n=30, you should indicate how many out of the total i.e. the fraction forming the percentage e.g. 70% (378/540) in L172;

***Response:*** *amended as suggested*

L172/73: Say …more than 70% of samples were from female animals;

***Response:*** *amended as suggested*

You can break the narrative at line 181, present the table and proceed;

***Response:*** *amended as suggested*

L182: Figure 3 is not about sero-prevalence in the provinces. Correct and place the figure after L182;

***Response:*** *amended as suggested*

Present Figure 4 at line 187 and mention it in the text;

***Response:*** *amended as suggested*

L196: Rephrase to avoid two brackets into each other;

***Response:*** *amended as suggested*

L236: Retain the citation format prescribed by the journal, throughout the manuscript;
Where the p values are very small, e.g. 8.0x10-7 in L189 etc., indicate them as p<0.001

***Response:*** *amended as suggested*

L235-253: Do not just repeat your results and compare them to those of neighbouring countries. Discuss what the implications are;

***Response:*** *amended as suggested*

L257-259: Is irrelevant;

***Response:*** *deleted as suggested*

L268-271: Not a conclusion from your results;

***Response:*** *deleted as suggested*

L273: You have no results indicating that most goats were not vaccinated and therefore cannot discuss

***Response:*** *amended as suggested*
